# Address on Domestic Health

**Published:** 1882-04

**Authors:** Alfred Carpenter

**Affiliations:** Croydon


### Selections.

An Address on Domestic Health. Delivered at the Annual Meeting of the Sanitary Institute in Brighton, December 16th. By Alfred Carpenter, M. D., Croydon.

Mr. President: My theme this morning is "Domestic Health," or a consideration of the means by which individual health is raised or lowered, and the period of the life lengthened or shortened by domestic means. That individual life is uncertain, is a truism which every one acknowledges; and yet, in the aggregate, its value is known almost to a day. The statistician can tell to a positive certainty how many among a given number of men, women and children, *pallida mors* will call his own within the next twelve months, provided the area be large enough, and the habits and character of the population understood. If a man take his wife and family to live in a particular district—say in Liverpool, Manchester, or some other commercial center—there is a certainty that their chance of living a certain number of days is less than one-half of that which would belong to them if they came to live at Brighton. If a man determine that his sons shall become publicans or grocers (with wine license), or shall go into any business which brings them constantly into contact with the manufacture or sale of intoxicating liquor, he takes away from the lives of those sons a number of years which would be theirs if he put them into the trade of a gardener, or sent them into the Church as parish priests. We know of some of the reasons for this result. They are clear and unmistakable. Then, again, taking aggregates of children collected together: there are, or were, some streets in Liverpool in which ninety out of every one hundred of the children who were born into the world died within the first five years of his existence; whilst the children belonging to the class of people, taken from their wretched homes and sent to such schools as those at Anerly, or the Beddington Female Orphan Asylum, have a mortality of about 3 to 1,000 *per annum.* In the one case, 900 die out of a 1,000 in five years, in the other only 15. The conditions which produce this state of things are well known; they are absent in many places; I will mention one class in particular—Her Majesty's convict prisons. In those select and exclusive establishments, the mortality is about 8 in 1,000; but, then, they are selected lives—lives picked out of the most unhealthy and disreputable classes of society. If the same rules as to health and manner of living could be observed by a similar number of persons taken from healthy stocks, and from individuals who had not damaged their constitutions by crime and debauchery, this death-rate would be still lower; 6.5 to 7 in the 1,000 is probably the normal mortality which would arise in such communities; and although we must not compare adults with children, yet a public health which should show such results would, in the course of years, add materially to the number of monogenarians in the death-list, and, in course of time, bring man up to the limit which has been placed by Providence to life on earth, viz., 100 years. I believe every person is entiiled to live to that age; and if he do not, he is deprived of a portion of his birthright, either by his own act, or by the act of other persons. There is a time when a man must go down to the grave, but it ought not to be in consequence of disease, and it ought not to be before he had passed at least ninety years on earth. Death will come, though disease need not; and death without disease would probably be more painful to men than the fact of being born into the world is to the child. Uneasiness there may be, but not that acute suffering which arises, in most instances, from a disobedience to God's laws, either on the part of the individual himself, or of the commonwealth to which he belongs, or of his predecessors or forebearers on earth.

What is disease? It is a departure from the condition of the body called health. When does health cease and disease begin? I believe that there is a border line in which the conditions necessary for the establishment of disease must have time to produce their results before the disease actually arises. It is in this border line that the work of the sanitarian is most manifest, and is most beneficial to the community, by preventing functional disturbances and the consequences which naturally follow from the loss which sickness produces. I am sometimes asked if there is anybody living in perfect health at this particular moment. That is a question which is difficult to answer, if by health we are to understand that there is to be no disturbance of the equilibrium of function in the body. It is probable that, on this basis, there is no one living among civilized beings who is perfectly healthy, but I contend that there are great numbers in whom the border line of the disturbance of equilibrium has not been passed, and in whom disease has not yet appeared. A knowledge of, and obedience to, the laws which regulate health and prevent disease is of the greatest importance to these persons, for unhealthy home-influences will prevent them throwing off their burthen. They are not well, but they are not ill. Conflict is going on between two forces, and sanitary or unsanitary conditions may decide the issue. Disease works its way in many directions, and by a thousand and one means. There are some classes of disease which arise from the influence of morbid poisons which can produce themselves in the human body; and in these cases, each person suffering from that particular form of complaint becomes a manufactory of disease-producing particles. If these be brought into contact with other beings, they spread that particular form in geometrical ratio. There are some people, however, who are able to resist the effect of this poison, either because they have already suffered from the disease in question, and are protected from its future consequences, or because some unknown condition is present which takes away their liability to suffer from it. This class of disease is called zymotic; it includes small-pox, fevers, cholera, diphtheria, *et id genus omne.* Some individuals and some families are much more susceptible to be influenced by this class of diseases than others, and some die much more rapidly than others from their effects. The causes for these differences are not difficult to understand. The places in which such diseases will be most fatal, when the particles capable of producing them ar introduced there, are pretty well known. The conditions of pro ducing fatality are of man's making, and can be removed by the action of man. What are those conditions? In a few words, they are due to those changes which flow from the natural result of the act of living.

The natural products of secretion, or the excreta which are either retained too long within the body itself, or being excreted, are not passed on to the vegetable kingdom for utilization and re-application to man's wants. These excreta are the mainsprings of this class of disease. They are allowed to remain where they ought not to be. It would be as impossible for zymotic diseases to exist among us as it is for fish to live long out of water, if all excreta were rapidly removed and immediately utilized. The presence of zymotic diseases in our midst is evidence that some kind of excreta is retained somewhere in too close proximity to particular individuals, for it to be safe for those persons to continue in contact with it. We are accustomed to regard excreta as simply matters which everybody naturally passes away, or ought to pass away, into our sewers. Some of them used to be stored in cess-pools. That fatal error has been abandoned in most civilized places in Great Britain, but people now think that, if excreta be sent into sewers, all is done which is requisite for safety to health. Let the destructive effects of foul air upon our *employés* at the Government offices, and the death of Dean Stanley, answer this suggestion; let the *Times* newspaper be consulted any day in the week, and you will find in the obituary a record of some one who has been cut off by active disease, the consequence of impurity in the house, which impurity is at present scarcely suspected to have been a cause for his death. It is hidden in the neighborhood of the home, and is possibly unknown. Erysipelas or carbuncle, or so-called inflammation of some particular organ, has cut off the victim in the prime of life, and the death is at present seldom referred to its real cause, viz., foul air in the house. Any sewer which smells offensively is wrongly constructed, and is antagonistic to the public health; sewers ought not to smell; fresh sewerage does not smell, and a sewer should be as clean as a back-kitchen sink.

There is no difficulty about this cleanliness, if sanitary engineers would understand the first principles of sanitary work.

The moment any kind of excreta has passed from the body, at that moment, or as soon as temperature is reduced, there is a cessation of those actions which give rise to offensiveness. The offensiveness produced within the body is not injurious to health under ordinary conditions, otherwise every person would rapidly poison himself. That offensiveness is gaseous, and is soon dissipated, and there is a border line of time for removal before any kind of dangerous offensiveness can arise. When it is produced, it is a proof that the excreta have been kept within range of human life too long. The offensiveness is caused by germs of molecules, capable of reproduction in a different form from those which previously existed; they are material particles, and not gaseous, which have grown in the excreted matters outside the body. They are new cultivations, and are not capable of doing serious mischief in the tissues of living persons. They are comparatively harmless within the body, until they undergo a kind of change of nature, which enables them to do things which they could not have done without that change, and which would not have taken place if there had not been something at hand to enable them to grow. This change is promoted by rest in contact with nitrogenous matters, and is opposed by motion. There are some natural conditions which entirely prevent that change in the dangerous direction. Those natural conditions are found in our mother earth, and in the growth of vegetable matter upon that earth. The changes are promoted by rest, and the atmosphere which arises in the presence of organic matter in a state of rest, viz., an atmosphere containing excess of carbonic acid. This excess is sure to increase when organic matter is changing without an abundant and continually new supply.

The first principles, therefore, of sanitary work are embodied in the immediate and direct removal of all excreta from our midst, and its conveyance to, and utilization or destruction in, those districts which Providence has designed for its reception. Six to twelve or twenty-four hours are required, according to temperature and season, before mischief can arise; but this margin of time is ample for its removal.

But some of my hearers may say, are not diseases infectious, and capable of being transmitted from one to another, irrespective of outside sanitary work? I have to answer yes to this question; unfortunately they are; but their infective character arises from the fact I have already mentioned—viz., there is defective sanitation at home. There is scarcely a house in the kingdom in which excreta are not to some extent retained. The most civilized and luxurious home is, in some cases, carefully prepared for the cultivation of disease-germs or factors, if they come into our midst; carpets, curtains, and comforts of all kinds retain the *debris* from our skins and our pulmonary membranes; the excreta from our sweat-glands are allowed to settle upon our uncleaned windows, out-of-the-way cornices, useless ledges, and so-called architectural or upholstering ornaments; and our smoking friends spit about in a fashion which allows forcing-beds for disease germs to exist everywhere; so that, but for ventilation, the smoking carriages on our railroads would be very pest-houses, if disease-germs find admission to their floors; whilst our sensitiveness to draughts, and the way in which we heat our dining-rooms and offices, our public places of resort, our churches, and our drawing-rooms, provide a forcing-house for the growth of disease-germs which it seems almost impossible to disperse. I say almost impossible: this is only as regards the present time; for, with the growth of knowledge, there will be a gradual removal of causes; and, when the wholesale distribution of disease germs by water companies and impure sources of supply, impure food, and by sewers, is absolutely stopped, the private multiplication and spread of factors will be capable of being stamped out in a far easier and more satisfactory way than is now the case. Pure air, pure water, pure food, and temperate habits will diminish the amount of pabulum in which disease-factors can develop; and with those benefits we shall have a diminishing amount of impurity in the blood of individuals, which will gradually be effected in consequence of a more perfect knowledge of hygiene. This will render the blood and other tissues of most people so free from unnatural excreta, that, in the words of the Psalmist, "they need not be afraid of the pestilence which walketh in darkness, nor of the destruction which wasteth at noonday." (Psalm xci, v. 6.) This was a condition of things which was well known to the great Jewish lawgiver. Obedience to the sanitary laws which were laid down by Moses, is a necessary condition to perfect health, and to a state which shall give us power to stamp out zymotic diseases. If those laws were observed by all classes, the zymotic death-rate would not be an an appreciable quantity in our mortality lists. It is the duty of the State to prevent the wholesale or general distribution of zymotic disease; and if, with public spirit acting in the right direction, we have private action in every case in opposition to its distribution, there will be a great obstruction to the establishment of factors in our midst. That action must be directed by an intelligent knowledge of the fact that every person suffering from zymotic disease is a manufactory of particles of infective matter, which particles only require to be deprived of beds in which they can increase and multiply, and then zymotic diseases will altogether disappear as epidemics.

Let us keep this fact in mind: that it is the retention of excreta without the body, which are in a state of rest, and which are not brought into contact with soils and growing vegetable life, that allows the multiplication of disease particles able to infect humanity. But we may also remember that, if that humanity be in a proper state of health, the germs may find admission to the system, but will fail to find pabulum to set up disease; or the pabulum may be so trivial in amount, that the disease is of the mildest and least dangerous character.

The chemical constituents of the secretions and of the excretions are so varied and changing, that it is easy to see that the pabulum may be very various in character; and, as each kind of disease-particle will require a different kind of pabulum, it follows that in one case the disease may be severe, and in another slight, or different in character altogether; and that the effects of the disease will depend upon the quantity of material upon which it can grow. I am of opinion that there is an analogy between the growth and development of disease both within and without the body; that the causes can only increase and multiply at the expense of used-up matter which has filled its mission in the human body, and which has not been removed in proper time from the tissues of that body, or from the neighborhood of its producers; that this used-up matter would be harmless if kept within the body only so long as may be necessary for its proper discharge.

and a disease-germ finding admission would fail to find food in sufficient quantity to live upon, and would abort, become unfruitful, and be incapable of producing evil; that it is the quantity of this pabulum which regulates the intensity of an attack of zymotic disease, assisted to some extent by the quantity or size of the dose of infective matter. A small dose takes considerable time to reproduce disease-product in sufficient amount in the tissues of the body to seriously interfere with general health, unless the pabulum be very great; but, when the dose is large, there is a more rapid reproduction, the stage of incubation is more decided, and the new crop, or new association of matter which the crop sets up, is capable of arresting some of the various functions of the body, which functions are necessary for the continuance of life. They interfere with the work of a gland, or take up material for their own nourishment which the body requires for healthy action; and so disease of organs arises which may or may not have a fatal effect, according to the amount of interference which they produce. If, however, the products of the act of life be removed from the body as soon as formed, or as soon as nature requires them to be removed, the germs of disease may be admitted without material risk; but, being admitted, the result of their action will be manifest according to the quantity of *débris* which is present for them to feed upon. They then grow, come to maturity, reproduce the minutest of the minute forms of perverted protoplasm, and, having fulfilled their mission, die a natural death. These diseased protoplasmic particles have to be expelled from the body (in small-pox, they form the pustules); and the causes of all the mischief break up into other organic forms, and become excreta from the body if health be restored. If, however, death take place, they continue to multiply after a short time in the tissues of the victim, until putrefaction has thoroughly broken up the animal matter into its simple elements. This is very easy to understand, when we remember that man produces in himself, by his own act of living, three or four of the most virulent poisons which can be manufactured. We are apt to think of the saliva of a mad dog, the secretion connected with the poison-fang of a snake, or the cause which produces the plague, as terrible things, to be kept a distance, and to be run away from at the shortest possible notice; and yet we manufacture in our own bodies, at every moment of our lives, poisons which are as virulent as any I have mentioned, or which can be produced anywhere in nature. The carbonic acid which we expire is instantly fatal when it is inhaled in its concentrated form. The cholesterin, or some products resulting from a change in the elements which form that material, and which is produced by nerve-action in our own persons, has only to be kept within the body, in the way which does happen sometimes in some forms of liver disease, to be fatal in a few hours. The same result follows in kidney maladies, in which the kidney refuses any longer to perform its work; and, if the skin be varnished over with some impermeable varnish, an end comes to life within a few hours, from the retention of an excretion which should be made by the skin, the retention of which is also the cause of fatal effects in some eruptive diseases. These excreta exist in some shape or other in the blood, and ought to be removed as rapidly as nature designed that they should be; and yet, by our domestic habits, by our taste for stimulants, by our absurd fashions, and by our so-called civilized life, we impede the actions of those organs which excrete those poisons; we keep the products of metamorphosis of tissues about our own persons, and then wonder that nature rebels at such treatment, and sets up actions of her own antagonistic to the deadly effects which such treatment is certain to produce. It is in consequence of these antagonistic actions, to the retention of these morbid poisons, that, as an ultimate result of that sickness, the mortality of our country, as well as of all others in the world, is so much higher than it ought to be.

It is a curious and a wonderful fact, that the usual effect of the self-manufactured poisons is not painful; deaths by carbonic acid, or urea, or acholia, are comparatively painless. Pain is the outcry of those watchful sentinels which Providence has given us for our protection; it is the danger-signal to be carefully considered, by means of which the approaching mischief may be warded off. The tendency of treatment by the medical profession at the present day is rather to ease pain, than to prevent a recurrence of those evils which give rise to pain. I trust, however, that a more sensible era is approaching, and that human foresight will take the direction which I have indicated—viz.: that of rather bearing with the pain and taking measures to prevent a continuance of its cause, than of rushing for relief of the pain itself to those agents which make us still more blind to its consequences, while we let the cause go on. I refer here more especially to the use of intoxicating liquors for assisting in perfect digestion, relieving neuralgic conditions, and obtaining an artificial and delusive warmth. The relief is obtained at the expense of a paralyzing action set up in the watchful sentinels of danger. The organic and sensitive nerves, which are the danger-signals, are put out of gear; and the train goes on heedless of the mischief, which is now hidden from feeling and personal observation. By these means, organic diseases arise in the different organs of the body. Brain, heart, lungs, liver, and kidneys, or spinal column, ultimately break down, and life is shortened in a way and by a means which is little thought of by a majority of the victims; and the daily use of alcoholic drinks assists this more than any other known agent.

Time would fail me to deal with all the causes of disease which arise from a want of proper knowledge among the people of the first principles of domestic hygiene, and of the evils which follow from a non-removal of the products of excretion, and the retention within ourselves and our houses of those deadly poisons which are manufactured by the human frame in the act of living. I must, however, remark that, as regards the outside of our bodies, it is not likely that the best method of dealing with excreta will be generally used, until public opinion has put a stop to that vicious principle which is adopted, of paying our engineers and our architects for the work they perform according to its cost, viz.: by a commission upon the cost of the work done. The great object is to expend large sums in great works, whilst the minor details, which take up the most time and cause the smallest expenditure of capital, are relegated to the care of those who have to inhabit the house after it has been built, or to keep the sewers in order after the engineer.

As regards the individual, I am of opinion that, sufficient as vaccination is at present in protecting the masses from the effects of small-pox, the right course to be followed in the prevention the effects of infectious disease is not to be found in trying to bring into vogue new methods of vaccinating, for the prevention of cholera, diphtheria, and others of that class, but in the removing from our persons and from our habitations those *débris* which arise from the act of living, and the removal of which would render us proof against the evils caused by contagion. However much, therefore, I may admire the efforts of Pasteur to prevent the spread of infectious disease at the present time among our domestic animals, I believe it is not the direction in which we should look in the future with regard to ourselves, except so that we may draw the inference that, as it is by cultivation that a germ becomes deadly, so, by a counter-cultivation, its deadly character may be done away with, and the disease rendered harmless. I prefer to follow upon the lines connected with the latter rather than the former contingency; and if the anti-vaccinationists were as energetic in the promotion of sanitary work, which has for its object the utilization of our excreta, as they are in antagonism to vaccination, they would be doing some good in the world, and would assist to bring about a condition of things which would render vaccination an unnecessary operation.

[Dr. Carpenter next commented at some length on the results arising from want of ventilation in homes, and the retention of carbonic acid and other excretions exhaled from the lungs in the act of breathing; reference being especially made to its influence in the production of tubercular disease.]

If it were not for circumstances connected with poverty, the poor would be rapidly swept from the face of the earth by tubercular disease; but it happens, fortunately for them, that their houses cannot be kept entirely draught-proof. Wind and storm find their way into their lodgings, and diminish the evil by diluting it, and that which they consider a misfortune is a real blessing. For dilution of the poison is an impediment to its effect. This good fortune does not apply to the same extent to the houses of the rich; they, as well as the poor, take care to exclude fresh air, and take the greatest possible trouble to keep oxygen outside, and to retain carbonic acid and its concomitants in their living-rooms. Every step which is taken to ensure comfort adds to the danger. The old-fashioned open fireplace and imperfectly fitting windows kept the enemy at bay for a time; but the chimney-corner is gone; window-frames are made so tight that not a breath of fresh air can get in by their means; and carpets, curtains, and other so-called comforts, remove from them all its freshness. The introduction of gas for illuminating purposes adds very much to the evil; each ordinary gas-burner uses up as much oxygen as three people; and now the custom is to render the walls perfectly impervious to the transmission of air, and still greater evil naturally follows. The result is that people, especially luxurious people, live in an atmosphere in which there is a diminution of the required oxygen, with increased amount of carbonic acid, with its concomitant excreta, which in themselves are still more injurious, as being food for debased protoplasm; and then they wonder why they are out of health; why they constantly take cold when exposed to draughts; why they lose their appetite; why they cannot sleep; and why they suffer from headaches and listlessness—why, in fact, they do not enjoy life, and feel thankful to the God who made them for the blessings which might be theirs, if they understood the laws which belong to the act of living.

Suppose we look into the church where we go to give God thanks for our many blessings. A thousand people are there, each occupying a square yard of floor-space. It may be even less than this, if it be a fashionable place of worship. The seats are cushioned, and the floor at the least matted. If it be cold weather, the great object of the verger is to keep the church warm and comfortable. The temperature is carefully observed to be above temperate on the thermometer, which hangs on the center pillars. If the air in that church were examined, it would be found to be already deficient in oxygen. Instead of 20.90, it is probably 20.70. A thousand people are assembled; each of these, on the average, produces .6 cubic feet of carbonic acid per hour, or a cubic foot during each ordinary service. A thousand cubic feet of a deadly poison are exhaled into the place; and, if there were no ventilation at all, the whole of the congregation would be suffocated before the service is over, for three per cent. of carbonic acid would soon be fatal. Each person uses up 16.6 cubic feet of air per hour; and, to keep down the level of carbonic acid to its natural standard, one hundred times that quantity of fresh air is required to be admitted for each individual. Dr. Parkes calculated that 2,800 cubic feet per hour per individual of fresh air were necessary to effect this object, beside the quantity required for the supply of gaslights, which may be used, and which are used in most places of public assembly, in the way which is most likely to damage the health of those exposed to its influence—viz.: by naked burners. The thousand people require 3,000,000 cubic feet of fresh air each hour to keep the poison within the natural limits, and at least half that amount to keep it within a safe amount. Is there any architect in the kingdom who provides this? Is there any architect in the kingdom who ever considers the subject from this point of view? The result of this neglect is the establishment of draughts, which the short-sightedness of people generally will not permit. They prefer to be poisoned by an anæsthetic painless process, rather than submit to discomfort and possible pain, with a diminution of danger.

There is also another result which follows from close packing: each person is a furnace, and thus tends to raise the temperature of the place in which the people are assembled to a point as near to 98.5° as the assembly will permit. This is a law established by Providence to prevent suffocation; for, as soon as there is a rise of the temperature of the air within the room, there is an immediate effort made by the outer air to bring it to an equilibrium. The higher the temperature, the more rapid the movement of the outer air to enter, and the more decided the draught. There is at once a cry, on the part of those exposed to the draught, to close the aperture, and check the benevolent designs of Providence, when the real indication afforded by the draught is that more openings are wanted. The offending opening is closed, with the certainty of greater injury being inflicted upon all the occupants of the room than it was possible for the draught to effect.

It ought to be an established ordinance, that architects should be called upon to provide entrance for fresh air, as well as exits for foul air, which will correspond with the number likely to be present in the building; and we ought to call upon all those who have the care of places of public resort, that there shall be an intelligent supervision of these means; that it shall not be left to an ignorant attendant to regulate the ventilation of a place for a cold night, and alter it as temperature rises inside or falls without. Each of these conditions ought to be watched, and provision made according to the requirements of each place; and, until these conditions are considered by architects, it will not be possible to prevent people assembling in great nnmbers from, to some extent, poisoning each other. As to our dwelling-houses, when the public shall come to understand that pain is an evidence of some wrong conduct on our own part, or on the part of some other persons toward us—induced, it may be, by regulations for the prevention of the ingress of fresh air into the house in which we live—architects will see that provision is made for free ventilation of a character which shall not destroy by imperfect designs; and they will not obey the wrong instincts of our nature, by trying to shut out the vivifying influence which pure and frequently renewed air alone can afford.—*British Medical Journal.*

---

SMALL-POX IN RICHMOND.—Rumors are wild as to the estimated number of cases of small-pox in this city. At the time of our last issue, there were not over seventy-five cases of small-pox and varioloid in the city. Now there are not as many as thirty-five—so much for vaccination. Every day the number of cases is decreasing, until now there is not the slightest ground for fear of its further material spread. The city Board of Health is not only vigilant in regard to this matter, but it is now clothed with sufficient authority to stamp out the disease entirely, and in a week or two after the issue of this December number, we venture the assertion that new cases will be very rare and far between.—*Virginia Med. Monthly.*

